# Repurposing Drugs for Mayaro Virus: Identification of EIDD-1931, Favipiravir and Suramin as Mayaro Virus Inhibitors

**DOI:** 10.3390/microorganisms9040734

**Published:** 2021-03-31

**Authors:** Lana Langendries, Rana Abdelnabi, Johan Neyts, Leen Delang

**Affiliations:** Laboratory of Virology and Chemotherapy, Rega Institute for Medical Research, Department of Microbiology, Immunology and Transplantation, KU Leuven, 3000 Leuven, Belgium; lana.langendries@kuleuven.be (L.L.); rana.abdelnabi@kuleuven.be (R.A.)

**Keywords:** Mayaro virus, antivirals, alphaviruses, emerging viruses

## Abstract

Despite the emerging threat of the Mayaro virus (MAYV) in Central and South-America, there are no licensed antivirals or vaccines available for this neglected mosquito-borne virus. Here, we optimized a robust antiviral assay based on the inhibition of the cytopathogenic effect that could be used for high-throughput screening to identify MAYV inhibitors. We first evaluated different cell lines and virus inputs to determine the best conditions for a reliable and reproducible antiviral assay. Next, we used this assay to evaluate a panel of antiviral compounds with known activity against other arboviruses. Only three drugs were identified as inhibitors of MAYV: β-D-N^4^-hydroxycytidine (EIDD-1931), favipiravir and suramin. The in vitro anti-MAYV activity of these antiviral compounds was further confirmed in a virus yield assay. These antivirals can therefore serve as reference compounds for future anti-MAYV compound testing. In addition, it is of interest to further explore the activity of EIDD-1931 and its orally bioavailable pro-drug molnupiravir in animal infection models to determine whether it offers promise for the treatment of MAYV infection.

## 1. Introduction

The Mayaro virus (MAYV) is a neglected, mosquito-borne virus that belongs to the alphavirus genus within the *Togaviridae* family [1]. MAYV circulates in a sylvatic cycle between non-human primates and forest canopy mosquitoes, including *Mansonia* sp., *Psorophora* sp. and *Haemagogus* sp. (mainly *Haemagogus janthinomys*). Accidental spillover to humans who are working or walking in the forests has been reported [2]. Experimental studies have shown that the anthropophilic *Aedes aegypti*, *Aedes albopictus* and *Anopheles* sp. might be competent vectors of MAYV as well, which could cause a spillover to the urban cycle in the future, resulting in more human cases [3,4]. The first MAYV strains were isolated from the blood of infected humans in Trinidad and Tobago in 1954. Since then, MAYV was repeatedly detected in humans and mosquitoes in Latin America and in the Caribbean [5,6]. In total, 901 cases have been reported so far, but many cases might be undetected since MAYV diagnostics are not widely available and cases may be clinically misdiagnosed as dengue (DENV) or chikungunya virus (CHIKV), as these viruses can induce similar symptoms and circulate in the same regions [6,7].

MAYV-induced disease consists of an acute and a subacute phase. Common symptoms during the acute phase are fever, maculopapular rash, headache, dizziness, chills, retro-orbital pain, myalgia, vomiting and diarrhea. More than 50% of the infected patients develop a subacute, long-term disease, characterized by a debilitating arthralgia that can persist for weeks to months [2,8]. Despite the emerging threat of MAYV, there are currently no licensed vaccines available to prevent or treat MAYV-induced disease. Four MAYV vaccine candidates have been developed in a preclinical stage: a formalin-inactivated virus vaccine [9], two live-attenuated virus vaccines [10,11] and a synthetic DNA-based vaccine [12], all inducing neutralizing antibodies and showing efficacy against lethal MAYV challenge in mice.

No specific antiviral drugs are available against MAYV infection. Analgesics and nonsteroidal anti-inflammatory drugs are used to relieve the pain [8]. Several small molecules (thienopyridine derivates [13], cerulenin [14], brefeldin A [15], prostaglandin A1 [16], bovine lactoferrin [17], ribavirin [13,18,19,20]) and compounds extracted from plant material (silymarin [21], epicatechin [18], quercetin [19]) have been described to have antiviral activity against MAYV in cell culture. Although these molecules have in vitro anti-MAYV activity, they are not very potent nor do they specifically target MAYV. Furthermore, none of these have progressed towards in vivo studies. There is thus a need to identify new compounds with antiviral activity against MAYV. Therefore, we optimized a robust antiviral assay based on the inhibition of the cytopathogenic effect, which could be used to screen extensive panels of molecules to identify MAYV inhibitors. In this assay, we assessed the effect of a small panel of antiviral compounds with known activity against other arboviruses and identified EIDD-1931, favipiravir and suramin as in vitro inhibitors. 

## 2. Materials and Methods

### 2.1. Cells and Viruses

African green monkey kidney cells (Vero cells, ATCC CCL-81) were maintained in Minimal Essential Medium (MEM, Gibco, Merelbeke, Belgium) supplemented with 10% fetal bovine serum (FBS, Gibco), 1% L-glutamine (Gibco), 1% sodium bicarbonate (Gibco) and 1% non-essential amino acids (NEAA, Gibco). Human skin fibroblasts (ATCC CRL-2522) were maintained in MEM medium supplemented with 10% FBS, 1% L-glutamine, 1% sodium bicarbonate, 1% sodium pyruvate (Gibco) and 1% NEAA. Baby hamster kidney (BHK) cells were maintained in Minimal Essential Medium REGA 3 (MEM Rega 3, Gibco) supplemented with 10% FBS, 1% L-glutamine and 1% sodium bicarbonate. Human hepatocyte-derived carcinoma (Huh-7) cells were cultured in Dulbecco’s Modified Eagle Medium (DMEM; Gibco) supplemented with 10% FBS, 1% NEAA, and 2% 4-(2-hydroxyethyl)-1-piperazineethanesulfonic acid (HEPES; Gibco). Adenocarcinoma human alveolar basal epithelial (A549) cells were maintained in DMEM supplemented with 10% FBS, 1% sodium bicarbonate and 1% sodium pyruvate.

All cell cultures were maintained at 37 °C in an atmosphere of 5% CO_2_ and 95%–99% relative humidity. Virus propagation and in vitro assays in all cell types were performed using similar media but supplemented with 2% FBS (assay media).

MAYV strain TC625 was obtained via the EVAg consortium (catalog number 001v-EVA502; https://www.european-virus-archive.com, accessed on 29 September 2017). The virus stock was propagated in Vero cells and was stored at −80 °C. The virus titer was determined by end-point titration and plaque assay on Vero cells.

### 2.2. Compounds

2′-*C*-methylcytidine (2’CMC) and 7-Deaza-2′-*C*-methyladenosine (7DMA) were purchased from Carbosynth (Berkshire, UK). Favipiravir (T-705) was purchased from BOC Sciences (Shirley, New York, USA). CHVB-032 was synthesized in the laboratory of Prof. G. Pürstinger (University of Innsbruck, Austria) and Prof. T. Langer (University of Vienna, Austria). MADTP-0372 was a kind gift of Prof. M.J. Pérez-Pérez (University of Madrid, Spain). Galidesivir, remdesivir and EIDD-1931 (β-D-N^4^-hydroxycytidine) were purchased from Medchem Express (Monmouth Junction, New Jersey, USA) and chloroquine from Sigma-Aldrich (Overijse, Belgium). Ribavirin, 1-(β-D-ribofuranosyl)-1H-1,2,4-triazole-3-carboxamide (Virazole) was obtained from ICN Pharmaceuticals (Costa Mesa, CA, USA), suramin sodium was purchased from Santa Cruz Biotechnology (Heidelberg, Germany) and arbidol was provided by Prof. S. Polyak (University of Washington, Seattle, WA, USA). 2’CMC, ribavirin, galidesivir, remdesivir and EIDD-1931 were dissolved at a concentration of 10 mM, favipiravir and 7DMA were dissolved at a concentration of 30 mM, CHVB-032 was dissolved at a concentration of 10 mg/mL and MADTP-0372 at a concentration of 5 mg/mL in analytical grade dimethyl sulfoxide (DMSO; Sigma-Aldrich). Chloroquine was dissolved in sterile milliQ water at a concentration of 30 mM. Suramin was dissolved in phosphate-buffered saline (PBS; Gibco) at a concentration of 10 mM. Arbidol was dissolved in absolute ethanol (VWR, Leuven, Belgium) at a concentration of 10 mg/mL and then 1:10 diluted in sterile milliQ water. The compounds were stored at 4 °C. All stocks were diluted in 2% FBS assay medium for experimental use.

### 2.3. Replication Kinetics

Vero cells were seeded in 96-well plates (BD Falcon, Erembodegem, Belgium) at a density of 5 × 10^4^ cells/well in 2% FBS assay medium and were allowed to adhere overnight in the incubator. The next day, cells were infected with MAYV at multiplicity of infection (MOI) 1 for 1 h. After the incubation time, the virus inoculum was removed, the cells were washed three times with PBS and fresh assay medium was added to the wells. At several time points (1 h, 4 h, 8 h, 16 h, 24 h, 48 h and 72 h) post infection (pi), culture supernatants were collected to determine extracellular infectious virus titers, quantified by end-point titration, and extracellular viral RNA, quantified by qRT-PCR.

### 2.4. End-Point Titration Assay

Vero, BHK, Huh-7, CRL2522 and A549 cells were seeded in 96-well plates (respectively at a density of 2.5 × 10^4^, 1 × 10^4^, 7.5 × 10^3^, 1.2 × 10^4^, 7.5 × 10^3^ cells/well) and were allowed to adhere overnight. The next day, 3 parallel 10-fold serial dilutions of the virus samples were prepared in the plates. After 3 days of incubation, the cells were examined microscopically for virus-induced cytopathogenic effect (CPE). A well was scored positive if any traces of virus-induced CPE were observed compared to the uninfected controls. The TCID_50_/mL was calculated using the method of Reed and Muench [22] and is defined as the virus dose that would infect 50% of the cell cultures. To determine the best cell line and the optimal MOI for the CPE reduction assay, cell viability was assessed using the MTS/PMS method as described by the manufacturer (Promega, Leiden, The Netherlands). Z’ values were calculated to determine the robustness of the assay in the selected cell lines. The Z′ value considers the range of the assay signal and signal variability [23]. We considered a Z′ value ≥ 0.7 as robust for high-throughput screening.

### 2.5. Plaque Assay

Vero cells were seeded in 6-well plates (BD Falcon, Erembodegem, Belgium) at a density of 1 × 10^6^ cells/well in 10% FBS containing MEM medium and were allowed to attach overnight at 37 °C and 5% CO_2_. The next day, the medium was aspirated and cells were washed with 2% FBS assay medium and cells were incubated with dilutions of virus samples in assay medium for 1 h at 37 °C. After incubation, the virus was removed and cells were washed three times with assay medium. The medium was discarded and a 1:1 mixture of 1% low-melting agarose (Invitrogen, Thermo Fisher Scientific, Erembodegem, Belgium) and pre-warmed 2× overlay medium (2× MEM medium (Gibco) supplemented with 4% FBS, 2% L-glutamine and 2% sodium bicarbonate) was added and was allowed to solidify completely. Plates were incubated at 37 °C for 3 days. After 3 days, cells were fixed with 8% paraformaldehyde (Sigma-Aldrich) for at least 2 h at room temperature. The agarose overlay was then removed and the fixed cells were washed three times with PBS, after which the cells were stained with crystal violet (Sigma-Aldrich) for 15 min. Cells were rinsed with water and plaques were counted visually to calculate the virus plaque-forming units (PFU) per mL.

### 2.6. Antiviral CPE Reduction and Cytotoxicity Assay

Vero cells were seeded at a density of 2.5 × 10^4^ in 96-well tissue culture plates and were allowed to adhere overnight in the incubator. The next day, dilution series of the compounds were prepared in 2% FBS assay medium, after which the cultures were infected with MAYV (MOI 0.01). At day 3 pi, the inhibition of CPE was quantified using the MTS/PMS method. The cells were checked microscopically for minor signs of virus-induced cytopathogenic effects or compound-induced adverse effects on cell monolayer morphology. The 50% effective concentration (EC_50_), which is defined as the concentration of compound that is required to inhibit virus-induced cell death by 50%, was determined using logarithmic interpolation. In parallel, the toxicity of the compounds was determined by exposing uninfected cells to the same serial dilutions of the compound used in the antiviral assay. At day 3 pi, the 50% cytotoxic/cytostatic concentration (CC_50_), i.e., the concentration of compound that is required to reduce cell viability by 50%, was determined microscopically or by using the MTS/PMS method.

### 2.7. Virus Yield Assay

Vero cells were seeded in 96-well tissue culture plates at a density of 5 × 10^4^ cells/well and were allowed to adhere overnight at 37 °C. The next day, cells were treated with serial dilutions of the compound and then infected with MAYV (MOI 0.01). After 2 h of infection, the cells were washed to remove non-adsorbed virus, followed by incubation with the same serial dilutions of compounds in fresh 2% FBS assay medium. After 48 h of incubation, supernatants were collected and viral RNA was isolated using the NucleoSpin RNA virus kit (Macherey Nagel, Eupen, Belgium) according to the manufacturer’s protocol. Viral RNA was quantified by qRT-PCR, while the infectious virus progeny was determined by end-point titration assay (TCID_50_/mL) as described before.

### 2.8. Quantitative Reverse Transcription PCR (qRT-PCR)

The sequences of primers used in qRT-PCR were: forward primer: 5′-TGGACCTTTGGCTCTTCTTATC-3′, reverse primer: 5′-GACGCTCACTGCGACTAAA-3′ (Integrated DNA Technologies (IDT), Leuven, Belgium) [24]. The probe sequence was 5′-/56-FAM/TACTTTCCTGCTGCAAGGGCTCTT/3BHQ_1/−3′ (IDT) [24]. One-step, quantitative RT-PCR was performed in a total volume of 25 µL, containing 13.94 µL H_2_O, 6.25 µL master mix (Eurogentec, Seraing, Belgium), 0.375 µL of forward primer, 0.375 µL of reverse primer (final concentration of each primer 150 nM), 1 µL of probe (final concentration 400 nM), 0.0625 µL reverse transcriptase (Eurogentec) and 3 µL RNA sample. The qRT-PCR reaction was performed with the Applied Biosystems 7500 Fast Real-Time PCR System (Branchburg, NJ, USA) using the following conditions: 30 min at 48 °C, 10 min at 95 °C, followed by 40 cycles of 15 s at 95 °C and 1 min at 60 °C. For quantification, standard curves were generated each run using 10-fold dilutions of MAYV viral RNA isolated from the virus stock.

### 2.9. Delay of Treatment Assay

Vero cells were seeded in 96-well plates at a density of 5 × 10^4^ cells/well and incubated overnight. Two hours prior to MAYV infection, 25 µM EIDD-1931, 300 µM favipiravir and 500 µM suramin were added to the cells. Subsequently, at time point 0, the medium was removed from all the wells and the cells were infected with MAYV (MOI 1), where after compounds were added at time points −2 and 0. After an incubation of 1 h, viral inoculum was removed, cells were washed 3 times with 2% FBS assay medium and compounds were again added at time points −2 and 0. Next, 25 µM EIDD-1931, 300 µM favipiravir and 500 µM suramin were added at 2, 4, 6 and 8 h pi. At 10 h pi, supernatants were collected to quantify infectious virus particles by end-point titration. Cells were washed 3 times with PBS and intracellular RNA was isolated by incubating cells for 15 min with 50 µL cell-to-cDNA lysis buffer (Ambion, Austin, TX, USA) at room temperature, where after cells were collected and incubated for 15 min at 75 °C. Viral RNA was 1:4 diluted with RNase-free water (Promega) and viral loads were measured by qRT-PCR. 

### 2.10. Statistical Analysis

All data were analyzed using GraphPad Prism 8.3.1. The results of the virus yield assays and delay of treatment assays were statistically analyzed using the Kruskal–Wallis test (nonparametric one-way ANOVA). Statistical significance threshold was assessed at *p* values < 0.05. Statistical details are described in the figure legends.

## 3. Results

### 3.1. Selection of Cell Line for MAYV-Induced Cytopathogenic Effect

**Figure 1:**
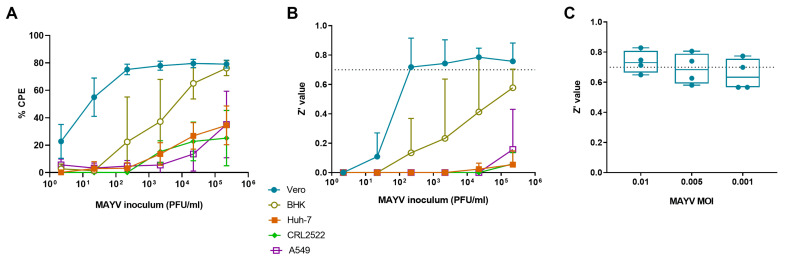
Optimization of a CPE-reduction antiviral assay. (**A**) Percentage CPE and (**B**) Z’ value per MAYV inoculum in different cell lines (Vero, BHK, Huh-7, CRL2522 and A549 cells). Results were obtained with the MTS/PMS read-out method. Mean values ± standard deviations (SD) are shown of 3 independent experiments. CPE, cytopathogenic effect; PFU, plaque-forming unit. (**C**) Z’ value for three different MAYV inocula (MOI 0.01, 0.005, 0.001) on Vero cells. Data are presented as box plots showing individual and mean values of four independent experiments. MOI, multiplicity of infection.

We infected several cell lines (human: skin fibroblasts, A549 and Huh-7 cells; monkey: Vero cells; hamster: BHK cells) with MAYV to explore whether the virus can induce clear signs of CPE that can be scored visually at day 3 pi. The CPE was most prominent in Vero cells, even at lower virus inoculum (still 55% CPE at an inoculum of 22 PFU/mL) (Figure 1A). A clear CPE developed also in BHK cells but only when a high virus inoculum (at least 2.2 × 10^4^ PFU/mL) was used. In Huh-7 cells, A549 cells and skin fibroblasts, MAYV did not induce a clear CPE. Z’ values were calculated; only for Vero cells the average Z’ value was higher than 0.7, even when the virus inoculum was low (220 PFU/mL) (Figure 1B). Based on these results, Vero cells were selected for the further development of an antiviral assay to identify MAYV inhibitors.

Next, the replication kinetics of MAYV TC625 in Vero cells were determined. A steep increase in viral infectious particles and viral RNA in the supernatant was observed between 4 h pi and 8 h pi (Supplemental Figure S1A). At 24 h pi, a plateau was reached. MAYV TC625 resulted overall in a uniform plaque phenotype in Vero cells (Supplemental Figure S1B).

### 3.2. Selection of Optimal Virus Inoculum

To identify the optimal virus inoculum for antiviral assays in Vero cells, the cells were infected with different MAYV titers to select the inoculum that resulted in the best Z’ value at day 3 pi. Figure 1C shows that a MOI of 0.01 resulted in a Z’ value higher than 0.7, whereas lower MOIs were slightly lower than 0.7.

### 3.3. Anti-MAYV Activity of a Panel of Reference Compounds

Next, we studied whether a panel of molecules that are known to have antiviral activity against other arboviruses [CHIKV, DENV, Zika virus (ZIKV), Yellow fever virus (YFV)] was also able to inhibit MAYV replication in Vero cells. The panel consisted of molecules with different mechanisms of action: arbidol [25] and chloroquine [26] inhibit viral entry; MADTP-0372 [27] and CHVB-032 [28] inhibit the capping of alphavirus RNA; suramin is able to block early steps of the virus life cycle [29,30]; ribavirin acts via indirect (depletion of GTP pools and immunomodulatory effects) or direct mechanisms (inhibition of viral replication and viral capping) [31]; favipiravir [32,33,34], 7DMA [35], 2’CMC [36], EIDD-1931 [37,38], galidesivir and remdesivir [39] are inhibitors of viral genome replication. 

**Figure 2:**
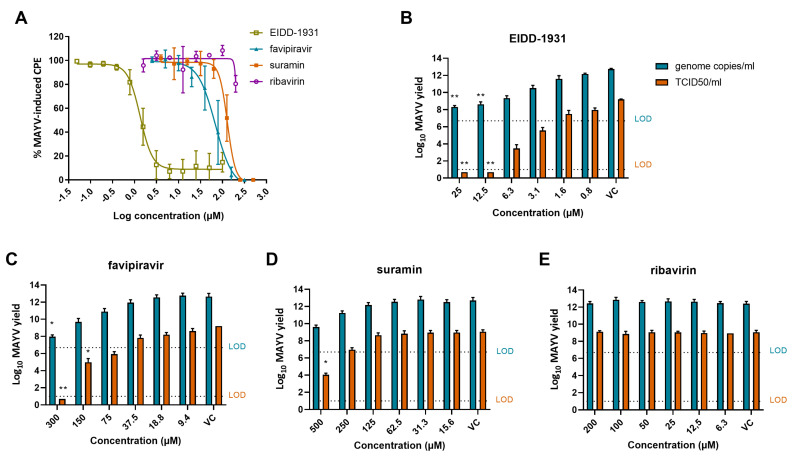
In vitro antiviral effect of EIDD-1931, favipiravir, suramin and ribavirin in Vero cells. (**A**) Dose–response effect of EIDD-1931, favipiravir, suramin and ribavirin on MAYV-induced CPE (MOI 0.01), quantified in Vero cells by the MTS/PMS method. Data shown are mean values ± standard deviations (SD) from three independent experiments. (**B**–**E**) The effect of different concentrations of (**B**) EIDD-1931, (**C**) favipiravir, (**D**) suramin and (**E**) ribavirin on the release of MAYV particles by infected Vero cells (MOI 0.01). Both viral RNA (genome copies/mL; blue) and infectious progeny virus (TCID_50_/mL; orange) were quantified at 48 h pi by real-time qRT-PCR and end-point titrations, respectively. Data shown are mean values ± SD from three independent experiments. Significant differences from untreated virus control were analyzed by Kruskal–Wallis test (*, *p* < 0.05; **, *p* < 0.005). CPE, cytopathogenic effect; TCID, tissue culture infectious dose; VC, virus control (untreated); LOD, limit of detection.

The EC_50_ and CC_50_ values of the different compounds were determined by colorimetric read-out and calculated using logarithmic interpolation (Table 1). Inhibition of MAYV-induced CPE was observed only when infected cells were treated with EIDD-1931, favipiravir or suramin (Figure 2A) with EC_50_ values of 1.6 µM, 79 µM and 124 µM, respectively. Other compounds of the panel were not active against MAYV (e.g., ribavirin) (Figure 2A).

### 3.4. EIDD-1931, Favipiravir and Suramin Reduce MAYV Yield

To confirm the results obtained with the optimized CPE reduction assay, a virus yield assay was performed, determining both extracellular infectious virus and viral RNA levels, using end-point titration and qRT-PCR, respectively. A dose-dependent reduction in viral RNA and infectious virus content was observed with increasing concentrations of EIDD-1931 (Figure 2B), favipiravir (Figure 2C) and suramin (Figure 2D). Viral replication was completely blocked (~8 log_10_ decrease in infectious virus) by EIDD-1931 upon a concentration of 12.5 µM and by favipiravir at the highest concentration tested (300 µM). An inhibitory effect was still observed for EIDD-1931 and favipiravir at 3.1 µM and 75 µM, respectively. Suramin resulted in a ~5 log_10_ decrease in infectious virus at the highest concentration tested (500 µM). In contrast, ribavirin was not effective in blocking MAYV infectivity (Figure 2E).

### 3.5. Mechanism of Action of EIDD-1931, Favipiravir and Suramin against MAYV

A delay of treatment assay was performed to elucidate at which stage of the replication cycle the compounds act. To this end, EIDD-1931 (25 µM), favipiravir (300 µM) or suramin (500 µM) were added at different time points and supernatants and cells were collected after a single replication cycle.

**Figure 3:**
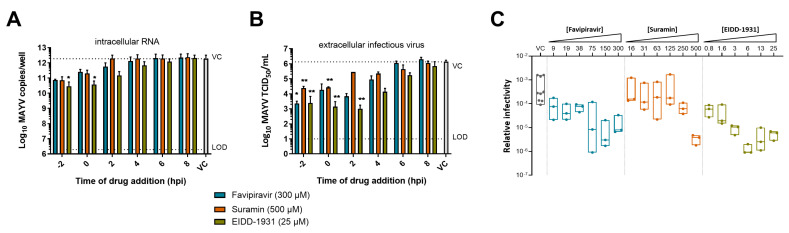
Mechanism of action of EIDD-1931, favipiravir and suramin against MAYV. Delay of treatment effect of favipiravir (blue), suramin (orange) and EIDD-1931 (green) on (**A**) intracellular RNA and (**B**) infectious virus titers, determined at 10 h pi, using qRT-PCR and end-point titrations, respectively. Vero cells were infected with MAYV TC625 (MOI 1) and compounds were added 2 h prior to infection (−2), at the time of infection (0) or 2, 4, 6, 8 h pi. Data shown are mean values ± SD from three (favipiravir and suramin) or four (EIDD-1931) experiments. Significant differences from untreated virus control were analyzed by Kruskal–Wallis test (*, *p* < 0.05; **, *p* < 0.005). (**C**) Relative infectivity (i.e., the ratio infectious virus: viral RNA) of MAYV in the supernatant in the presence of different compound concentrations. Data are presented as floating bars showing median values of three virus yield experiments. TCID, tissue culture infectious dose; VC, virus control (untreated); hpi, hours post infection; LOD, limit of detection.

The reduction in intracellular RNA levels gradually decreased upon time of addition of the compounds to the infected cell cultures (Figure 3A). Favipiravir and suramin reduced intracellular RNA only when added at early time points (2 h prior to infection and 0 h pi), whereas EIDD-1931 still resulted in a reduction when added until 2 h pi. Infectious virus titers decreased by suramin when added before or at the time of infection (~−2 log_10_; Figure 3B). Favipiravir, on the other hand, reduced viral titers until 2 h pi (~2–3log_10_ reduction). Further treatment delay resulted in a gradual loss of antiviral activity for both compounds. EIDD-1931 caused a pronounced decrease in infectious virus until 4 h pi (~2–3 log_10_ reduction), but still showed modest activity when added at 6 h pi. These data suggest that suramin acted at early stages of the viral life cycle, whereas EIDD-1931 had prolonged antiviral activity and operated during the stage of viral RNA synthesis. Favipiravir, on the other hand, inhibited MAYV infection at a stage that coincides with the onset of viral RNA synthesis.

Lethal mutagenesis is the mechanism by which EIDD-1931 [40] exerts its antiviral effect and it is also stated as one of the antiviral mechanisms of favipiravir [32]. To assess whether this is also true for their activity against MAYV, we calculated the relative infectivity (i.e., the ratio of infectious virus vs. viral RNA) of MAYV in the supernatant in the presence of different concentrations of EIDD-1931, favipiravir and suramin (Figure 3C). A significant decrease in relative infectivity was observed starting from 6 µM of EIDD-1931 (~1000-fold) and 75 µM of favipiravir (~100-fold). In contrast, the relative infectivity of MAYV was not affected by suramin, with the exception of the highest concentration tested (500 µM). These data suggest that EIDD-1931 and favipiravir inhibit MAYV infection in cell culture by inducing error-prone replication. 

## 4. Discussion

Several arboviruses have become global health threats in the past decades. Due to increased travelling, climate change and adaptation of the arthropod vectors to urbanization, the geographic distribution of arboviral infections has expanded and is still expanding in many regions of the world [41]. A medically important arbovirus of the alphavirus genus is CHIKV, which has been spreading worldwide the past few years. CHIKV was first described during an outbreak in Tanzania in 1953 and was further identified during several outbreaks in Africa and Asia [42]. In 2007, CHIKV transmission was reported in Europe, and in December 2013, it was introduced into the Americas [43]. To date, human CHIKV cases have been detected in more than 100 countries [42]. Approximately 60% of the patients develop persistent arthralgia and arthritis, resulting in decreased quality of life for months to years following initial infection [44]. Lessons should be learned from these CHIKV outbreaks and preventive measures should be introduced to control outbreaks with neglected arboviruses. Such a neglected arbovirus is MAYV, which can cause a disease comparable to that of CHIKV in humans [1]. Experimental studies have shown that the anthropophilic *Aedes* mosquito spp. could be competent vectors for MAYV [4], which may possibly lead to a more global spread of the virus. To be prepared for a possible MAYV outbreak, we should arm ourselves with potent antivirals to prevent and treat MAYV-induced disease.

For this purpose, it is essential to have an unambiguous antiviral assay that is able to be used for screening of extensive compound libraries to identify potent MAYV inhibitors. In vitro activity of several small molecules or extracted plant material against MAYV has been demonstrated by performing a virus yield assay [13,16,19,20,21]. Although reliable, this is a highly time-consuming and/or expensive assay which cannot be implemented to test large libraries of compounds. Nevertheless, virus yield assays are still needed to confirm antiviral activity of compounds that were picked up from phenotypic screens. Recently, an antiviral screening assay for MAYV was developed based on an eGFP reporter MAYV which is suitable for high-throughput screening, in contrast to virus yield assays [45]. Caution has to be taken since reporter viruses can be attenuated or face issues of stability. We optimized a reproducible and reliable antiviral assay, based on CPE reduction, using the MTS/PMS read-out method and using the MAYV strain TC625. This method has been developed before for other viruses, such as CHIKV [46] and DENV [47], but was now optimized for MAYV.

We used this assay to evaluate the antiviral activity of a panel of antivirals with known activity against other arboviruses (including entry, capping, replication inhibitors), hereby creating a reference framework for future anti-MAYV compound testing. EIDD-1931, favipiravir and suramin were identified as inhibitors of MAYV-induced CPE in Vero cells. These compounds were originally used against other viruses: EIDD-1931 was developed to treat influenza [48], but is also active against SARS-CoV-2 infection, for which it is tested in clinical trials (phase II/III) [49]. Favipiravir is an influenza drug that has been approved in Japan for the treatment of pandemic influenza virus [50], and suramin is used for the treatment of African sleeping sickness and river blindness [51]. Favipiravir and suramin showed only modest activity against MAYV (EC_50_ values of 79 µM and 124 µM, respectively), whereas EIDD-1931 was found to be a potent inhibitor with an EC_50_ value of 1.6 µM. In our hands, ribavirin was not active against MAYV using a starting concentration of 200 µM. This concentration was selected since signs of toxicity were determined microscopically at higher concentrations. The lack of antiviral effect of ribavirin in our study is in contrast to previous work [13,19,20] in which high EC_50_ values ranging from 62.5 µM [13] to 255 µM [19,20] were reported in virus yield assays. This discrepancy might be due to the use of a different virus strain (TR4675 [13,19,20]) or virus inoculum (MOI 0.05 [13] or 0.1 [19,20]). The capping inhibitors MADTP and CHVB were not active against MAYV. Previously, it was shown that both molecules were specifically acting against CHIKV in cell culture-based assays and were not able to potently inhibit other alphaviruses such as Semliki Forest virus and Sindbis virus [27,28]. Remdesivir [52], galidesivir [53], 7DMA [35] and 2’CMC [36] were not able to inhibit MAYV either. It has been previously described that these compounds inhibit flaviviruses, but lack activity against alphaviruses. Chloroquine and arbidol did not show activity against MAYV, while they have proven antiviral activity against CHIKV (EC_50_ values of ~10 and 12 µM, respectively [25,26]).

We determined whether previously described antiviral action mechanisms of EIDD-1931 [40], favipiravir [32,33,34,54] and suramin [29,30] were responsible for the antiviral effect against MAYV. Our data suggest that EIDD-1931 and favipiravir inhibited MAYV at the replication stage through lethal mutagenesis, similar to what has been reported for CHIKV [38,55] and Venezuelan equine encephalitis virus [40]. EIDD-1931 was acting at later time points than favipiravir, which is probably due to the fact that favipiravir needs to be metabolically activated into favipiravir ribofuranosyl 5′-triphospate, which requires several hours to achieve effective concentrations [56]. Suramin has been identified to inhibit a post-attachment step of CHIKV, likely viral entry [30]. The delay of treatment results of this study showed that suramin acted during the early steps of the MAYV life cycle, suggesting that a similar mechanism of action is possible. 

In conclusion, we established a robust in vitro antiviral assay for MAYV and identified three small molecules with antiviral activity (EIDD-1931, favipiravir and suramin) which can be used as references in future screening efforts. In addition, it would be of interest to further explore the effect of the orally bioavailable pro-drug of EIDD-1931 against MAYV infection in animal infection models.

## Figures and Tables

**Table 1:** EC_50_ and CC_50_ values of an antiviral compound panel evaluated against MAYV.

Compound	MOA *	EC_50_ (µM)	CC_50_ (µM)
Arbidol	Entry inhibitors [25,26,30]	>60	>100
Chloroquine		>200	145 ± 20
Suramin		124 ± 26	>2000
EIDD-1931	Virus replication inhibitors [32,35,36,38,39]	1.6 ± 0.5	>100
Favipiravir		79 ± 4	2837 ± 86
7DMA		>60	>600
2’CMC		>20	167 ± 7
Galidesivir		>100	238 ± 36
Remdesivir		>100	297 ± 101
Ribavirin	Replication inhibitor + host effects [31]	>200	250 ± 81
MADTP	Capping inhibitors [27,28]	>100	>400
CHVB-032		>100	>75

* MOA, mechanism of action: mechanism of the antiviral effect described for other alphaviruses.

## Data Availability

All data supporting the findings of this study are available in the main text and/or the supplemental information.

## Supplementary Materials

The following are available online at https://www.mdpi.com/article/10.3390/microorganisms9040734/s1, Figure S1: Viral fitness of MAYV in Vero cells.
