# Supplementary figures and images for: Repurposing Drugs for Mayaro Virus: Identification of EIDD-1931, Favipiravir and Suramin as Mayaro Virus Inhibitors
Source: Microorganisms. 2021 Mar 31;9(4):734. doi: 10.3390/microorganisms9040734 (PMC8065421; doi:10.3390/microorganisms9040734)

**A**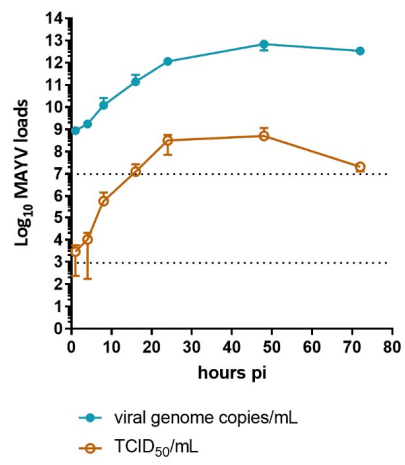**B**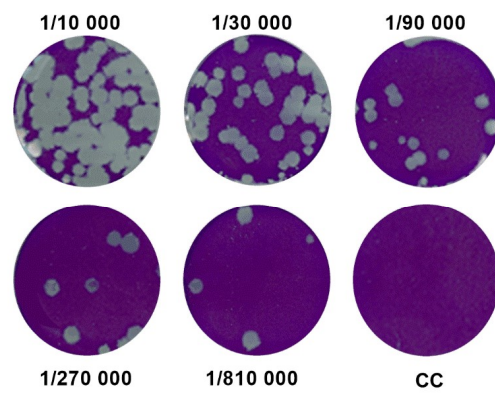

**Figure 1.** Viral fitness of MAYV in Vero cells.

Supplement: Supplementary file 1 [file microorganisms-09-00734-s001.pdf]
